# Addressing and proposing solutions for unmet clinical needs in the management of myeloproliferative neoplasm-associated thrombosis: A consensus-based position paper

**DOI:** 10.1038/s41408-019-0225-5

**Published:** 2019-08-08

**Authors:** Tiziano Barbui, Valerio De Stefano, Anna Falanga, Guido Finazzi, Ida Martinelli, Francesco Rodeghiero, Alessandro M. Vannucchi, Giovanni Barosi

**Affiliations:** 1 FROM Research Foundation, Papa Giovanni XXIII Hospital, Bergamo, Italy; 2 Fondazione Policlinico Universitario A. Gemelli IRCCS, Roma, Italy; 3 Istituto di Ematologia, Università Cattolica, Roma, Italy; 4 Department of Immunohematology and Transfusion Medicine and the Haemostasis and Thrombosis Center, Papa Giovanni XXIII Hospital, Bergamo, Italy; 5 University of Milan Bicocca, Milan, Italy; 6 Hematology Division, Papa Giovanni XXIII Hospital, Bergamo, Italy; 7 Fondazione IRCCS Ca’ Granda Ospedale Maggiore Policlinico, A. Bianchi Bonomi Hemophilia and Thrombosis Center, Milan, Italy; 8 Hematology Project Foundation, affiliated to the Department of Hematology, San Bortolo Hospital, Vicenza, Italy; 9 CRIMM-Center of Research and Innovation of Myeloproliferative Neoplasms, Azienda Ospedaliera Universitaria Careggi, Dept Experimental and Clinical medicine, and Denothe Center, University of Florence, Florence, Italy; 10 Center for the Study of Myelofibrosis, IRCCS Policlinico S. Matteo Foundation, Pavia, Italy

**Keywords:** Myeloproliferative disease, Risk factors

## Abstract

This article presents the results of a group discussion among an ad hoc constituted Panel of experts aimed at highlighting unmet clinical needs (UCNs) in the management of thrombotic risk and thrombotic events associated with Philadelphia-negative myeloproliferative neoplasms (Ph-neg MPNs). With the Delphi technique, the challenges in Ph-neg MPN-associated thrombosis were selected. The most clinically relevant UCNs resulted in: (1) providing evidence of the benefits and risks of direct oral anticoagulants, (2) providing evidence of the benefits and risks of cytoreduction in patients with splanchnic vein thrombosis without hypercythemia, (3) improving knowledge of the role of the mutated endothelium in the pathogenesis of thrombosis, (4) improving aspirin dosing regimens in essential thrombocythemia, (5) improving antithrombotic management of Ph-neg MPN-associated pregnancy, (6) providing evidence for the optimal duration of anticoagulation for prophylaxis of recurrent VTE, (7) improving knowledge of the association between somatic gene mutations and risk factors for thrombosis, and (8) improving the grading system of thrombosis risk in polycythemia vera. For each of these issues, proposals for advancement in research and clinical practice were addressed. Hopefully, this comprehensive overview will serve to inform the design and implementation of new studies in the field.

## Introduction

Classical Philadelphia-negative myeloproliferative neoplasms (Ph-neg MPNs) include polycythemia vera (PV), essential thrombocythemia (ET), and myelofibrosis (MF). The discovery that driver somatic mutations (primarily *JAK2*V617F) and additional subclonal mutations contribute to the pathogenesis of Ph neg-MPNs^1^ has opened a new era in both the biological understanding of these disorders and the associated clinical practice. In addition, new advances in the knowledge of molecular pathogenesis have also improved our understanding of the pathogenesis of MPN-associated arterial and venous major thrombosis, and new data on the complex interplay among blood cells, endothelium and coagulation system are now available^2^. Therefore, major advances have been attained in terms of prognostic classification and, interestingly, new therapeutic hypotheses about thrombotic risk and events have been raised^3–5^.

Despite this increase in knowledge, arterial thrombosis and venous thromboembolism (VTE) remain the principal causes of morbidity and mortality in patients with Ph-neg MPNs^6,7^. Thrombosis can occur from the very early stages of the disease and complicates the course of the follow-up^6^ with an incidence of either arterial thrombosis or VTE approximately fivefold to tenfold higher than in the general population^6^. This increase is mostly because new concepts for thrombosis management were translated from a limited number of interventional clinical studies^8^.

In this review, we attempt to define the unmet clinical needs (UCNs) in Ph-neg MPN-associated thrombosis, which relate principally to an improvement in risk classification, trial design, and structured care delivery. For this aim, a group of experts was convened to participate in a special consensus project.

## Methods

A Panel of Italian experts (hereafter referred to as the Panel) was selected based on the individual expertise in research and clinical practice with Ph-neg MPNs. During an initial meeting on June 2018, the outline of the project was discussed, and the topics that form the structure of the present document were decided. The key UCNs were selected through a series of questionnaires according to the Delphi technique^9^. Afterward, each panelist drafted statements addressing one identified UCN, and the remaining panelists scored their agreement with those statements and provided suggestions for modifications. Finally, the Panel convened for a consensus conference held in Milano, Italy, in October 2018. At this conference, the final proposals were given using the nominal group technique^9^, by which participants including European experts and members of the European Leukemia Net (ELN), were first asked to comment on their disagreements with the proposed issues in round-robin fashion and then to vote for a final statement. The resulting general discussion provided remarkable input to the present report.

## Results

Although numerous UCNs in Ph-neg MPNs were issued by the Panel (Table 1), this review focuses on some of the major outstanding challenges voted as the most relevant and urgent by the panelists.

**Table 1:** Candidate unmet clinical needs (UCNs)

No.	Candidate UCNs
1	Providing evidence of the benefits and risks of DOACs in MPNs
2	Providing evidence of the optimal duration of anticoagulant therapy in patients with MPN and thrombosis
3	Increasing the evidence on efficacy of ruxolitinib as antithrombotic agents
4	Improving knowledge of the association between driver mutations and risk factors for thrombosis in MPNs
5	Testing the validity of surrogate endpoint for the experimental assessment of antithrombotic action of cytoreductive drugs in MPNs
6	Providing evidence of the benefits and risks of cytoreduction in patients with SVT thrombosis without hypercythemia
7	Providing evidence of the benefits and risks of JAK2 inhibitors in MPN-associated SVT
8	Improving knowledge on risk factors of recurrence of thrombosis in MPNs
9	Improving knowledge of the role acquired thrombophilia has on the development of thrombosis in MPNs
10	Improving knowledge of the time from an MPN-associated thrombotic event and diagnosis of MPN
11	Improving knowledge of the role of mutated endothelium in the pathogenesis of thrombosis in MPNs
12	Improving knowledge of the leukocyte number as a risk factor for thrombosis and bleeding in MPNs
13	Improving the diagnostic pathway of MPNs in the setting of splanchnic vein thrombosis
14	Improving the diagnostic pathway of MPNs in the setting of cerebral vein thrombosis
15	Optimizing the use of Willebrand test for assessing the risk of bleeding in extreme thrombocytosis in MPNs
16	Improving the use of primary antithrombotic prophylaxis in patients with myelofibrosis
17	Improving the dosing regiments of aspirin in ET
18	Improving antithrombotic prophylaxis in MPN pregnant patients

### UCN1: providing evidence of the benefits and risks of direct oral anticoagulants in Ph-neg MPNs

In the general population, the direct oral anticoagulants (DOACs) dabigatran, rivaroxaban, apixaban, and edoxaban are approved for treatment of VTE^10^, perioperative thromboprohylaxis, and ischemic stroke prevention in atrial fibrillation (AF)^10,11^. In these settings, DOACs are as effective as warfarin and have a lower rate of intracranial hemorrhage.

In the cancer population, two recent prospective RCTs of edoxaban^12^ and rivaroxaban^13^, versus low-molecular-weight heparin (LMWH) demonstrated the noninferiority of DOACs for VTE treatment in cancer patients.

In Ph-neg MPNs patients, VTE treatment with vitamin K antagonists (VKAs) provides good protection against recurrence, although rates of both recurrent thrombosis and major bleeding remain higher than in non-MPN patients^14^. DOACs can represent a suitable alternative, with the potential to protect both arterial and venous thrombosis and to perform better than VKAs in reducing bleeding risk^15^. Knowledge of VTE treatment with DOACs in Ph-neg MPN patients is limited. In the two aforementioned trials, only 10% of recruited patients had hematological malignancies. In the OBENE registry, of the 760 MPN patients included, only 13 patients were receiving DOACs for AF and 12 for VTE^16^. In the German MPN registry of the Study Alliance Leukemia, 68 of 454 patients had deep vein thrombosis (DVT) or splanchnic vein thrombosis (SVT), and only eight were treated with rivaroxaban^14^.

The major issue in planning clinical trials with DOACs in cancer patients is trial feasibility. In the Hokusai trial^12^, a sample size of ~1000 patients was required, whereas in the SELECT-D trial, a target sample size of 530 was necessary^13^.

### Consensus statements

*Evidence of the benefits and risks of DOACs for the prevention of VTE recurrence has been so far achieved with noninferiority study designs in the normal population and in cancer patients. A noninferiority design is considered reasonable when evaluating new treatments that offer greater convenience for patients while providing similar efficacy*.

*It should be highlighted that the most conservative noninferiority margin assumes that the upper limit of the two-sided 95% CIs of the hazard ratio is below the prespecified margin of 2.00. This margin sets the required sample size at approximately 1000 randomized patients*.

*In the setting of Ph-neg MPNs, which are rare diseases, the Panel agreed that a pragmatic pivotal randomized comparison of DOACs to the standard-treatment control, with the primary end-point defined as the recurrence of any VTE, could improve the trial’s feasibility*.

*The investigators hypothesized that the benefit/harm profile of DOAC treatment will be noninferior to, or better than, usual care with LMWH/VKAs among the MPN patients. The information gained will empower MPN patients and physicians to make more informed choices about anticoagulation strategies to manage VTE*.

*Assuming a one-year VTE-free survival of 90% (10% cumulative incidence) for both arms, if there truly is no difference between the standard and experimental treatment, then 284 patients are required to be 80% sure that the upper limit of a one-sided 97.5% confidence interval will exclude a difference in favor of the standard group greater than 10%. Allowing for 5% nonevaluable patients, a total of 300 patients need to be randomized*.

*However, the Panel agreed that while awaiting the results of such a trial, the prevention of recurrent VTE in MPN patients should be based on VKA unless some individual factors prompt using DOACs*.

### UCN2: providing evidence of the benefits and risks of cytoreduction in patients with splanchnic vein thrombosis without hypercythemia

Cytoreductive therapy, along with full anticoagulation, represents the standard of care for patients with Ph-neg MPN and SVT^17^. However, a large proportion of patients do not exhibit classical phenotypic blood counts for Ph-neg MPN at time of diagnosis (37.6 and 42.8% in recent multicenter cohort studies)^18,19^. The appropriateness of a myeloproliferation-targeted therapy in these patients is uncertain and has never been experimentally explored.

Approximately half of the *JAK2*V617F-positive SVT patients with a normal blood count at diagnosis will not develop an overt Ph-neg MPN during follow-up, and the clinical course of most of these patients is indolent^20,21^. Thus, a “watch-and-wait” approach, with cytoreduction starting only when a clear myeloproliferative pattern eventually appears, could be a reasonable approach.

The response to cytoreductive therapy in Ph-neg MPN patients with normal blood count is difficult to assess, since the current response criteria, developed for overt MPN, do not apply^22,23^. Two recently proposed SVT treatment algorithms in MPN do not recommend cytoreduction in patients with normal blood values^17,24^.

### Consensus statements

*The Panel agreed that the issue of benefits and risks of cytoreduction in patients with SVT without hypercythemia should be explored with an experimental approach*.

*The design of a randomized trial should address uncertainty in the simplest technical way, and the uncertainty principle should be used as the patient’s eligibility criterion*.

*The target of therapy should be the reduction of hematological parameters down to the limit of threatening cytopenia*.

*The primary endpoint of the trial should be recurrence of thrombotic events*.

*However, the Panel highlighted the critical conundrum that the necessity of a randomized design conflicts with the necessity of collecting a large number of patients for testing a clinically relevant statistical hypothesis*.

*The Panel agreed that a prospective observational study should represent a feasible and informative solution that could be a first step forward toward preliminary information and toward creating a network of participating centers for a formal clinical trial*.

### UCN3: improving knowledge of the role of mutated endothelium in the pathogenesis of thrombosis in MPNs

Two independent reports have opened a new avenue for investigating the role of endothelial cells (ECs) in the pathogenesis of Ph-neg MPN-associated thrombosis. In 2009, Sozer et al.^25^ reported that the ECs of two patients with Budd-Chiari syndrome and PV were homozygous for *JAK2*V617F. In 2013, Rosti et al.^26^ documented that splenic ECs harbored the *JAK2*V617F mutation in patients with MF bearing the mutation in their granulocytes. Subsequently, the detection of either *JAK2*V617F- or *CALR-*mutated ECs in the bone marrow (BM) of patients with MF showed that the phenomenon is diffuse and not limited to the *JAK2*V617F mutation^27^.

Although the origin of the mutated endothelium is still under investigation^28–30^, its biological implications are relevant due to the functional consequences of the mutation at a cellular level^31–35^, and the phenomenon could be the dominant cause of endothelial dysfunction in MPNs^36–41^.

Investigation into circulating and tissue-derived ECs is technically challenging, and contradictory results are frequently reported^42–47^. In cultures from BM, the main problem is overgrowth with pericytes or smooth muscle cells or contamination with fibroblasts, mesenchymal stromal cells and/or macrophages. However, it should be possible to set up cultures of ECs, free of non-EC contamination, by immunomagnetic depletion of monocyte/macrophages, followed by positive selection of the remaining cells with beads coated with antibodies directed toward EC-specific antigens (VE-cadherin, CD31, CD146) and using EC-specific medium and fibronectin or collagen-coated flasks. This approach is hindered by the limited availability of BM aspirates, due to the fibrotic marrow of MF patients, and the lack of a standard single cell isolation technique from BM biopsies. The very low amount of circulating ECs discourages the use of peripheral blood as the source for EC isolation and purification^48^.

### Consensus statements

*The Panel agreed that the phenomenon of mutated endothelium in MPNs needs to move from a “proof of principle” issue to a study of a high number of well-represented patients*.

*For field advancement, there is a need for a direct, ex vivo approach to study the human endothelium in a condition as close as possible to its in vivo natural environment*.

*Endothelial cell purification and culture is proposed as the method of election. BM EC culture has the advantage of giving easy and ethically sustainable access to the relevant diseased organs in patients at risk of or with thrombotic complications*.

*The Panel claimed that optimization and standardization of EC cultures from BM-derived cells should be an impelling objective for the research community*.

*This technique should be used to answer relevant questions emerging after the discovery of a mutated endothelium in Ph-neg MPNs, i.e., its frequency, distribution, and its functional and clinical significance*.

*The association of a mutated endothelium with splanchnic vein thrombosis is a prime research topic that can be approached with cross-sectional analysis of patients with or without a thrombotic event (case-control study)*.

### UCN4: improving the dosing regimens of aspirin in ET

No randomized trials have addressed the safety and efficacy of low-dose-aspirin (LDA) in ET such that, except for high-risk patients^49–52^, no definite data are available on its efficacy and safety, either alone or in association with hydroxyurea or anagrelide. The use of LDA in ET is justified based on indirect evidence and favorable results coming from the ECLAP study in PV^53^. However, based on new prognostic scores in ET, observation alone is considered a viable option for “very low-risk” patients (aged <60 years, without history of thrombosis and/or risk factors, and unmutated *JAK2V607F*). In absence of contraindications, all other patients are thought to benefit from LDA therapy. However, this indication does not apply to secondary prophylaxis in which LDA is clearly indicated^54^.

Single doses of 70–100 mg nonenteric-coated aspirin are not questioned, since even 75 mg is at least twice as high as the lowest dose necessary and sufficient to fully inhibit platelet COX-1 activity with repeated daily dosing^55^. However, preclinical evidence supports more frequent dosing due to accelerated renewal of platelet COX-1 secondary to the increased platelet production in ET, as indicated by the increased rate of immature platelets and by the measurement of serum TXB2^56,57^. Measurement of this surrogate biomarker of aspirin efficacy showed that platelets from 80% of aspirin-treated ET patients were inadequately inhibited by the traditional, once-daily, low-dose regimen^57^. Hence, extrapolation to ET patients of the 100-mg once-daily regimen, borrowed from PV and cardiovascular disorders, does not seem to be justified in light of altered aspirin pharmacodynamics in ET^55^.

A twice-daily regimen of 100 mg aspirin successfully improved the inhibition of serum TXB_2_ in a small, proof-of-concept study^58^, and this schedule is already advised in high-risk patients^59,60^. On the other hand, twice-daily dosing may reduce compliance, inhibit prostacyclin (PGI_2_) and increase bleeding.

### Consensus statements

*The Panel agreed that optimization of LDA for primary prophylaxis of thrombosis in ET requires randomized trials investigating once daily versus more frequent administrations of LDA to evaluate clinical outcomes in terms of thrombosis and bleeding*.

*However, the Panel also argued that the most appropriate aspirin dose and dosing interval should be identified before conducting such a trial. This dosing information could be attained through a properly sized, dose-finding study that should measure mechanism-based, surrogate biomarkers of efficacy and safety*.

*Serum TXB*_*2*_ *and urinary 2,3-dinor-6-keto-PGF*_*1a*_^60^ *are the most attainable biomarkers to test the adequacy of platelet COX-1 inactivation and sparing of endothelial COX-2, respectively, throughout a variable dosing interval of aspirin in the low-dose range. Recently, the design of a prospective study to find the most appropriate schedule of LDA based on biological biomarker modifications has been published and results are awaited soon*^61^.

### UCN5: improving antithrombotic management of Ph-neg MPN-associated pregnancy

Management of pregnant women with Ph-neg MPNs is challenging because both pregnancy and MPN increase the risk of thrombosis, bleeding and obstetrical complications. In the absence of acquired von Willebrand disease or platelet dysfunction, antithrombotic prophylaxis with aspirin or/and LMWH during pregnancy and puerperium is considered along with cytoreduction with interferon (safe for the fetus)^62,63^.

No randomized or controlled study on efficacy or safety of antithrombotic or cytoreductive treatment in pregnant women with MPNs is available. Available data stem from case reports, small case series, and retrospective monocenter and multicenter cohorts often pooled in reviews^64–74^. One prospective observational study in pregnant women with MPNs is available^75^, but considering its several limitations, it does not add more solid data. However, the complication rates were lower than previously reported. Successful pregnancy is 96% versus 60–80%, miscarriages are 1.7% versus 22–40%, and thrombosis is absent. The rate of pregnancy-related thrombosis in cohort studies is 11–15%^76,77^, but a meta-analysis reported an antepartum risk of 1.3% and 2.5% with and without LMWH, respectively, and a postpartum risk of 1.8% and 4.4% with and without LMWH, respectively^74^. Thrombotic risk appears higher in women with PV than ET^64–66^. The risk of bleeding (mainly postpartum hemorrhage) varies between 3 and 12.5%^74,76,77^. The rate of placenta-mediated pregnancy complications appears higher than in the general population. Fetal growth restriction is 3–22%, preeclampsia is 4–15%, and stillbirth is 1.5–6%^78^.

The role of the *JAK2*V167F mutation is controversial^66,79,80^. A platelet count >1000 × 10^9^/L, previous thrombosis, bleeding or obstetrical complications appear associated with an increased risk of obstetrical complications. Women with at least one of these predictors have a “high risk”, and may deserve a more aggressive antithrombotic prophylaxis^66,72^. Apparently, age, parity, leukocyte or platelet count, and hemoglobin levels do not have predictive value^66,75^.

Data on the management of pregnant women with PMF are limited to fewer than 20 case reports^81^.

### Consensus statements

*The Panel agreed that evidence of the benefits and risks of more aggressive antithrombotic prophylaxis in pregnant women with Ph-neg MPN should ideally be acquired through a prospective, randomized, controlled trial with maternal thrombotic events, obstetrical complications, and bleeding events as a composite endpoint. However, the Panel highlighted that such a study with a randomized design conflicts with the need to collect the high number of patients needed to test a clinically relevant statistical hypothesis. Moreover, the Panel underlined the ethical reasons that prevent randomization of patients to potentially dangerous therapies without preliminary evidence of benefit*.

*Retrospective observational studies, such as those with a case-control design comparing patients who receive an aggressive antithrombotic prophylaxis or standard care have possible biases (indication, exclusion bias) that make the resulting evidence of limited quality*.

*The Panel agreed that a prospective observational study should be feasible and could provide preliminary information leading to creating a network of centers for a formal clinical trial with hypothesis testing as if women were to be randomized. Investigators will clearly state the purpose or main hypotheses, type of treatment and outcomes, possible confounders, primary analyses and sample size*.

### UCN6: providing evidence of the optimal duration of anticoagulant therapy for prophylaxis of recurrent VTE

The rate of recurrent VTE in the general population is 5–7% pt-years^82^. After discontinuation of anticoagulation, the recurrence rate at 1 and 5 years is 10% and 30% after unprovoked VTE, respectively, and 5% and 15% after VTE provoked by nonsurgical reversible factors, respectively^83,84^. In non-MPN patients, these estimates drive the duration of VKA or DOAC treatment after VTE ranging from a minimum of 3 months to indefinite prolongation^9^.

Several retrospective studies estimated the rate of recurrent thrombosis in Ph-neg MPN patients. The incidence of recurrent thrombosis was estimated to be 7.6% pt-years^54^, 3.4 on VKAs and 9.4 off VKAs, respectively (*p* = 0.016)^85^. In a single-center study, the recurrent VTE rate was 6.0% pt-years, with more events off VKAs^86^. In 206 patients with well characterized diagnosis of DVT of the legs and/or PE, the incidence of recurrent thrombosis was 5.3% pt-years on VKAs and 12.8 after discontinuation (*p* = 0.008). After stopping VKAs, the recurrence rate was 42.3% at 5 years^87^. Patients with thrombosis of hepatic^18^ or cerebral veins^88^ were more prone to recurrences. Recurrent thrombosis can circumvent the effect of cytoreduction^54,85–87^, and recent reappraisal of data from retrospective cohorts and clinical trials showed that the efficacy of hydroxyurea in preventing thrombosis is significant for arterial sites but doubtful for venous sites^4,5^.

A major concern during anticoagulation is bleeding risk. In non-MPN patients receiving VKAs, the rate of major bleeding is 1.2–2.2%^89–92^. In Ph-neg MPN patients, the incidence of major bleeding is 0.9–2.4% pt-years on VKAs and 0.7–1.5 off VKAs and is as high as 2.8% pt-years when combining VKAs and aspirin^54,85,87^.

A recent survey reported high heterogeneity in treatment practices following thrombosis in Ph-neg patients^93^. Consensus statements and expert opinions suggested a prolongation of anticoagulation after unprovoked proximal DVT and/or PE, a life-threatening VTE or a VTE recurrence^94–96^, but there are no controlled studies addressing this issue.

### Consensus statement

*The Panel agreed that further knowledge about the relationship between the duration of anticoagulation and the occurrence of rethrombosis in Ph-neg MPN patients is needed in order to provide evidence supporting decisions on anticoagulant prophylaxis of recurrent VTE*.

*This knowledge may allow the identification of categories of patients at different risk of rethrombosis and of bleeding, exploring the role played by potential risk factors (e.g., previous unprovoked VTE, proximal DVT, pulmonary embolism, male sex, D-dimer level, residual vein thrombosis, thrombophilia) or disease-related biomarkers (e.g. JAK2V617F mutation, leukocytosis)*.

*To reach this goal, the Panel proposed a systematic review and meta-analysis of existing studies to summarize the evidence comparing various durations of secondary prophylaxis in Ph-neg MPNs. However, due to the literature being limited to cohort studies only, the pooled event rates and subgroup analyses will be the primary goal of such a meta-analysis*.

*The results could be the basis for a prospective observational study aimed to provide evidence for recommendations on duration of anticoagulant prophylaxis*.

### UCN7: improving the knowledge on the association between somatic mutations and risk factors for thrombosis in MPNs

An association of the *JAK2*V617F mutation and/or the variant allele frequency (VAF) with thrombosis was advocated in several retrospective analyses^97–99^. Patients with ET harboring the *JAK2*V617F mutation are at increased thrombotic risk, particularly if compared with *CALR* mutated or patients without known driver mutations (“triple negative”)^100,101^; these findings led to the development of the IPSET score that includes *JAK2*V617F mutation as a risk variable (HR 2.0)^3,102,103^. Conversely, *JAK2*V617F VAF is not currently included in risk scores for PV. Mechanistic explanations for the association of *JAK2*V617F with thrombosis are scant and are mainly based on biological plausibility. Sustained activation of JAK2/STAT signaling causes erythrocytosis, leukocytosis and, to a lesser degree, thrombocytosis. Increased red cell mass is a major determinant of thrombotic events, as far known^104^ and supported by the CYTO-PV study^105^; although not prospectively validated yet, leukocytosis is associated with increased thrombosis rate^106,107^. Abnormal *JAK2*V617F-mediated Rap1-GTPase activation in neuthrophils, resulting in increased VCAM1/ICAM1-mediated cell adhesion to the endothelium^108^ and neutrophil extracellular trap formation^109^, may mechanistically link mutated JAK2 to thrombosis pathogenesis. In addition, selected nondriver, myeloid neoplasms-associated mutations (in particular, *DNMT3A, TET2, ASXL1*) may underlie an increased risk of thrombosis, since CHIP (Clonal Hematopoiesis of Indeterminate Potential), was associated with atherosclerotic cardiovascular disease in elderly healthy subjects^110,111^.

### Consensus statements

*The Panel agreed that the most straightforward approach of defining the value of driver mutations as predictive risk factors for thrombosis in Ph-neg MPN should be a prospective study*.

*Although thrombosis may occur at a similar rate in PMF and ET, the complexity of clinical course and the confounding effects of therapies make PMF unsuitable for investigation; therefore, in principle, studies should be limited to patients with PV and ET*.

*One should accept the intrinsic variabilities of a “real-life” approach and perform prospective observational studies that include patients enrolled and genotyped at the time of diagnosis and followed according to the “best-practice”, until a predefined number of events is registered. An extensive database with all potential variables currently known to influence thrombosis rate should be used for multivariable analysis; accordingly, the number of patients to include may be indeed very high*.

### UCN8: improving the grading system of the risk of thrombosis in PV

Current risk stratification in PV is designed to estimate the likelihood of future arterial and venous thrombosis and considers low- and high-risk categories based on the respective absence or presence of either age >60 years or history of thrombosis. A matter of discussion is whether these definitions predicting thrombotic risk are still valid and whether the newly proposed disease-related risk factors, such as cardiovascular risk factors, leukocytosis, JAK2V617F allele burden, may improve the grading system of the thrombotic risk.

The Panel claimed that white blood cell (WBC) represents a clinically relevant candidate risk factor. As a matter of fact, the prognostic role of elevated WBC counts, but not thrombocytosis, was demonstrated in subanalysis of randomized clinical trials and in multicenter observational studies^63^. In a subanalysis of the ECLAP trial^106^, WBC > 17 × 10^9^/L was independently associated with myocardial infarction, and in patients of the high-hematocrit arm of CYTO-PV trial, the risk of thrombosis was clearly increased in the presence of WBC count above 6 × 10^9^/L, becoming statistically significant when WBC count was above 12 × 10^9^/L (hazard ratio, 4.89; 95% confidence interval (CI), 1.1–22.7; *P* = 0.04)^112^. Whether leukocytosis is a marker or a causative factor for thrombosis is a matter of discussion^113^.

### Consensus statements

*The Panel agreed that the major challenge in using leukocyte count as a risk factor for thrombosis in PV is the acquisition of strong evidence that a threshold value for leucocyte count represents a risk factor for thrombosis*.

*With such knowledge, experimental studies could be planned to evaluate thrombotic risks and benefits in terms of thrombotic event reduction with cytoreductive therapy*.

*The Panel proposed a comprehensive systematic review and meta-analysis of existing observational studies as the best method for summarizing the evidence comparing various event rates at different leukocyte counts*.

*The quality of the studies (good, fair and poor) should be assessed by awarding scores in each domain using a validated methodological evaluation tool, such as the Newcastle-Ottawa Scale (NOS), and publication bias should be properly evaluated*.

*The aims of this systematic review and meta-analysis should be to assess leukocyte count relative risk estimates associated with arterial and venous major thrombotic events in adult patients with PV, to verify the results of the meta-analysis in subgroups of patients with ET and PV, and to evaluate a possible WBC cut-off able to predict vascular complications*.

*The Panel agreed that the results of the meta-analysis could provide a basis for testing the prognostic accuracy of the best cut-off of WBC count in large, existing international registries, which could subsequently be incorporated into the grading system of thrombotic risk in PV and ET*.

## Conclusion

In this paper, UCNs with greater clinical relevance for Ph-neg MPN-associated thrombosis were identified, and consensus-based statements were issued by a Panel of experts and addressed mainly to the scientific community. Even though the molecular revolution that followed the discovery of the *JAK2*V617F mutation has opened several new research paths, of the eight selected UCNs, three addressed the diagnosis or risk stratification of thrombotic events. This decision argues that standardization of diagnostic pathways and patient risk stratification are prerequisites for any research on new therapeutics.

The major discussions about the proposed solutions for the UCNs were of methodological grade. As a matter of fact, many needs for Ph-neg MPN-associated thrombosis resulted from practical difficulties in increasing experimental knowledge in the field. This need mostly derives from the issue of the rarity of Ph-neg MPN patients, which urges designing nonrandomized studies with the power to provide new strong experimental knowledge. The Panel largely drew in a high number of methodological novelties proposed to solve the rarity problem, particularly with regard to the risk of selection and motivation biases^114–116^.

The hope is that this comprehensive overview will serve to inform the design and implementation of research in the field and will help the management of special clinical situations in these disorders.
